# Integral Valorization of Grape Pomace for Antioxidant Pickering Emulsions

**DOI:** 10.3390/antiox12051064

**Published:** 2023-05-08

**Authors:** Julen Diaz-Ramirez, Senda Basasoro, Kizkitza González, Arantxa Eceiza, Aloña Retegi, Nagore Gabilondo

**Affiliations:** Materials+Technologies’ Group, Engineering School of Gipuzkoa, Department of Chemical and Environmental Engineering, University of the Basque Country (UPV/EHU), Pza. Europa 1, 20018 Donostia-San Sebastián, Spain; julen.diaz@ehu.eus (J.D.-R.);

**Keywords:** agricultural residue, grape polyphenols, valorization, antioxidant, Pickering emulsion, sustainability

## Abstract

Full harnessing of grape pomace (GP) agricultural waste for the preparation of antioxidant Pickering emulsions is presented herein. Bacterial cellulose (BC) and polyphenolic extract (GPPE) were both prepared from GP. Rod-like BC nanocrystals up to 1.5 µm in length and 5–30 nm in width were obtained through enzymatic hydrolysis (EH). The GPPE obtained through ultrasound-assisted hydroalcoholic solvent extraction presented excellent antioxidant properties assessed using DPPH, ABTS and TPC assays. The BCNC-GPPE complex formation improved the colloidal stability of BCNC aqueous dispersions by decreasing the Z potential value up to −35 mV and prolonged the antioxidant half-life of GPPE up to 2.5 times. The antioxidant activity of the complex was demonstrated by the decrease in conjugate diene (CD) formation in olive oil-in-water emulsions, whereas the measured emulsification ratio (ER) and droplet mean size of hexadecane-in-water emulsions confirmed the physical stability improvement in all cases. The synergistic effect between nanocellulose and GPPE resulted in promising novel emulsions with prolonged physical and oxidative stability.

## 1. Introduction

An emulsion is a heterogeneous mixture of two or more normally immiscible liquids, referred to as oil-in-water (O/W) or water-in-oil (W/O) in the case of binary ones. Nowadays, the use of emulsions is fully widespread, mainly in the food and cosmetic industries, and, therefore, their stability is a critical issue that must be carefully controlled [1]. Indeed, the undesirable phenomena of phase inversion, flocculation and coalescence affect the physicochemical properties, and surfactants or stabilizing particles are necessary [2]. 

In this context, Pickering emulsions, in which surfactants are replaced by solid particles, have recently gained interest, since they reduce coalescence risks, add adjustable permeability and lead to unique rheological behavior [3,4]. The current trend in the preparation of Pickering emulsions lies in the use of greener stabilizing particles that are biodegradable and biocompatible, thus suitable for human intake or use [5].

In the last decade, bacterial cellulose (BC) has drawn the attention of the scientific community because of its low-cost, non-toxic character and its outstanding physicochemical properties [6]. Similarly, when BC is subjected to enzymatic hydrolysis (EH) or acid hydrolysis (AH) treatments, rod-shaped bacterial cellulose nanocrystals (BCNCs) can be obtained [7,8]. Although AH methods have advantages in terms of yield, it is necessary to look for greener and economically sustainable alternatives, such as EH [9]. 

BCNCs are distinguished by their high crystallinity, green condition and particularly high L/D aspect ratio. Therefore, BCNCs have great applicability in various fields, including the stabilization of Pickering emulsions [10,11,12]. The morphology and amphiphilic nature of BCNCs allow the stabilization of the oil–water interface of O/W emulsions at lower concentrations than other inorganic spherical particles used for Pickering emulsions [13]. Furthermore, as BCNCs are easily hydrated and well dispersed in aqueous media, they are capable of emulsifying a wide range of oils [12,14]. Cellulose nanocrystals (CNCs) can not only stabilize the prepared O/W emulsion, but they can also create synergistic complexes with other components in the emulsion to further improve stability and/or provide additional bioactivity [4]. The numerous free hydroxyl groups available in cellulosic chains enhance their reactivity and enable the formation of strong interactions with different molecules. In particular, the interactions between cellulose and different polyphenolic compounds have been tested to improve the physical and oxidative stability of lipids in O/W emulsions [15,16,17,18]. Polyphenol–cellulose-based complexes mostly interact through hydrogen bonding, hydrophobic and ionic interactions [19,20]. These interactions have been previously exploited for the encapsulation of curcumin or raspberry phenols to protect and extend their bioactive properties [21,22]. Furthermore, phenolic acids such as tannic acid have been shown to be effective in adjusting the hydrophobicity and increasing the applicability of CNCs in Pickering emulsions [4,16]. However, the literature related to the combination of cellulose and polyphenols in the application of Pickering emulsions is still scarce.

Polyphenolic extracts from plants have been proposed for colloidal aggregates intended for the food, pharma and cosmetic industries [23,24]. These polyphenolic compounds are especially abundant in lignocellulosic residues such as grape pomace (GP). GP is one of the most abundant agro-industrial wastes and it is composed of the remaining mixture of skins, stalks and seeds from grape pressing in the wine industry. In fact, polyphenolic extracts from GP (GPPE) have demonstrated antioxidant, anticancer, anti-inflammatory and antimicrobial activity, among others [25,26]. The antioxidant capacity of stabilizers aimed for emulsions is particularly important in the food or cosmetic industries, where the quality and nutritional value of lipids could be lost during storage. In fact, anthocyanins from black rice or epigallocatechin gallate from green tea have already proved to be useful in inhibiting lipid oxidation in emulsions [18,24]. In this regard, green technologies (supercritical fluids, microwaves or ultrasound) with low environmental impact and antioxidant activity preservation of the extracts are preferred [27]. 

In this work, the contribution of polyphenolic compounds to BCNCs stabilizing active Pickering emulsions through an integral harnessing concept of agroindustry wastes was assessed. With this aim, GP was used as a raw material for the biosynthesis of BC and, at the same time, as a source of polyphenolic extract after ultrasound-assisted hydroalcoholic treatment. BCNCs obtained through EH were evaluated as emulsifiers in terms of colloidal stability and antioxidant capacity, and their competitiveness with AH was assessed in different concentrations. The synergistic performance of the BCNC–polyphenolic extract complex was analyzed by monitoring the oxidative stability of olive oil-in-water and the physical stability of hexadecane-in-water Pickering emulsions.

## 2. Materials and Methods

### 2.1. Materials and Reagents

Yeast extract (≥89.0%), citric acid (C_6_H_8_O_7_ ≥ 99.5%), ascorbic acid (≥89.0%), cellulase from *Trichoderma* sp. 6,9 U/mg, 6-hydroxy-2,5,7,8-tetramethylchroman-2-carboxylic acid (Trolox), 2,2-diphenyl-1-picrylhydrazyl (DPPH), potassium persulfate (K_2_S_2_O_8_), Folin–Ciocalteu, sodium carbonate (Na_2_CO_3_) and 2,2'-azinobis(3-ethylbenzothiazoline-6-sulfonic acid) diammonium salt (ABTS) were purchased from Sigma Aldrich, and potassium hydroxide (KOH), sulfuric acid (H_2_SO_4_ ≥ 95%), acetic acid (HAc ≥ 99.5%) and sodium citrate buffer were obtained from Panreac Applychem (Spain). Methanol (99%) was acquired from Scharlau (Spain) and hexadecane from Merck (99%). Commercial olive oil and polysorbate 20 (Tween 20) from Qerlan were also used. GP culture medium and GPPE were prepared from Hondarrabi zuri white grape variety pomace, which was kindly provided by Bodega Butroi, a local winery (Biscay, Spain). 

GP was collected on the day of grape harvesting, after destemming and pressing the grapes under identical conditions. Finally, the GP, consisting of some stems, seeds and skins, was stored at −80 °C until its analysis.

*Komagataeibacter medellinensis* bacteria strain ID13488 (CECT 8140) was isolated from vinegar broth fermentation and kindly supplied by New Materials Research Group, Pontificia Bolivariana University of Medellín, Colombia [28]. 

### 2.2. BCNC Production

#### 2.2.1. BC Biosynthesis

BC membranes were biosynthesized by *K. medellinensis* in GP culture media under static growth conditions following the procedure previously reported by our group [29]. Briefly, GP was crushed with a blender and a 5% (*w*/*v*) juice was prepared with distilled water. The GP mixtures were passed through a cloth strainer and the filtrate was subjected to consecutive ultrasonic and hot water hydrolysis treatments. After this, the GP solutions were autoclaved and incubated with the bacteria at 28 °C for 13 days (1% *v*/*v* inoculum). The media were supplemented with yeast extract, and 250 mL Erlenmeyer flasks were used for incubation. The obtained BC membranes were washed with KOH at 2% (*w*/*v*) for 24 h, and afterwards, they were subjected to several wash cycles with deionized water until total neutralization. In Figure S2, a schematic representation of the entire experimental process carried out in this work can be observed.

#### 2.2.2. BC Hydrolysis

For the EH, the procedures detailed in [30] were conducted with some modifications. BC membranes were cut into pieces, surface water was removed and they were mechanically disintegrated with a blender at maximum speed to yield a final uniform paste. The paste was then subjected to a high-shear homogenization (Ultraturrax IKA T25, Staufen, Germany) for 10 min at 15,000 rpms. Then, 20 g of this BC paste was mixed with 200 mL of 0.1 M sodium acetate buffer (pH 5.2 ± 0.1). The mixture was kept overnight at 55 °C under gentle agitation; thus, the buffer completely infiltrated the BC network in order to facilitate the subsequent enzymolysis [31]. Then, 10 mg of cellulase (*Trichoderma reesei* 6.9 U/mg) was added, and the reaction was maintained at 55 °C and 300 rpm magnetic stirring for 4 days. The final cloudy solution was placed in a cold water bath to slow down the reaction. The resulting solution was centrifuged three times at 4500 rpm for 15 min (Hettich Zentrifugen D-78532, Tuttlingen, Germany). The precipitate was then thoroughly washed with cold distilled water to remove the buffer and residual enzyme, freeze-dried (Telstar LyoQuest HT40) and stored in a desiccator until use. The BCNCs obtained from EH are referred to as BCNC EH in this work. 

Similarly, the used AH method was adapted from that published by Yan et al., 2017 and George et al., 2012 [12,30]. First, 20 g of BC paste was mixed with 120 mL of 50% (*v*/*v*) sulfuric acid. The hydrolysis process was carried out for 4 h at 80 °C at 300 rpm magnetic stirring. Cold distilled water was added to stop the hydrolysis at a 1:5 (*v*:*v*) ratio with respect to the reaction volume. The resulting solution was subjected to three 15 min centrifugation cycles at 4500 rpm. The precipitate was suspended in cold distilled water after each centrifugation cycle, and it was ultrasonicated (JP Selecta 3,000,683 50/60 Hz, Barcelona, Spain) for five minutes. Finally, the obtained suspension was dialyzed against distilled water (Medicell Membranes Ltd., 3.5 KDa, London, UK) to a neutral pH. The BCNC suspension was freeze-dried and maintained in a desiccator until use. The BCNCs obtained from the AH method are referred to as BCNC AH in this work.

The EH and AH yields were calculated gravimetrically related to freeze-dried untreated BC weight following Equation (1):

(1) $$Hydrolysis\;yield\;\left(\%\right)\;=\;\frac{W_{BCNC}}{W_{BCp}}\;\cdot \;100$$

where *W_BCNC_* is the weight of the freeze-dried BCNCs and *W_BCp_* is the estimated freeze-dried weight of the BC paste used in each hydrolysis reaction.

### 2.3. BCNC Physicochemical and thermal Characterization 

#### 2.3.1. Atomic Force Microscopy (AFM)

Atomic force microscopy (AFM) images of BCNC samples were obtained in tapping mode using a Nanoscope IIIa scanning probe microscope (MultimodeTM Digital instruments, Bruker Corporation, Billerica, MA, USA) with an integrated force generated by cantilever/silicon probes, applying a resonance frequency of about 180 kHz. Cantilevers with tips of 5–10 nm in radius that were 125 μm long were used. A BCNC/water suspension drop was applied on mica and spin-coated at 2000 rpm for 120 s (Spincoater SCC-200 K.L.M. Micromaterials GmbH, Frankfurt, Germany). Prior to imaging the sample in a vacuum chamber for 24 h at room temperature, water was removed. AFM software was utilized to obtain diameter and length measurements of the samples from height images.

#### 2.3.2. X-ray Diffraction (XRD)

XRD patterns of both the BC membranes and BCNCs were obtained using a PHILIPS X’Pert Pro diffractometer (Almelo, Netherlands) in θ—θ configuration with a secondary monochromator with CuKα (λ = 0.154 nm) and a solid-state pixel detector, operating at 40 kV with a filament of 40 mA. The diffraction data were collected from 2θ values of 5° to 40°, where θ is the angle of incidence of the X-ray on the sample.

The crystallinity index (CI) of produced BCNCs was determined using the following equation [32]:

(2) $$CI\;\left(\%\right)\;=\;\frac{\left(I_{200}\;-\;I_{am}\right)}{I_{200}}\;\cdot \;100$$

where *I*_200_ is the maximum intensity of the (200) lattice diffraction at 2θ = 22.7° and *I_am_* is the intensity scattered by the amorphous domain of the sample (the location of the amorphous material signal considered was at 2θ = 18°).

#### 2.3.3. Fourier Transform Infrared Spectroscopy (FTIR)

A Nicolet Nexus spectrophotometer (Thermo Fisher Scientific, Waltham, MA, USA) equipped with an MKII Golden gate accessory (Specac) with a diamond crystal at a nominal incidence angle of 45° and a ZnSe lens was used. Spectra were recorded between 4000 and 650 cm^−1^, averaging 32 scans with a resolution of 4 cm^−1^.

#### 2.3.4. Thermogravimetric Analysis (TGA)

The thermal degradation of the BCNCs was monitored by using a TGA/DSC3 + Mettler Toledo analyzer (Greifensee, Switzerland). Samples of about 5 mg were submitted to a 10 °C min^−1^ heating ramp from 25 to 800 °C in a nitrogen atmosphere in order to prevent thermo-oxidative degradation.

#### 2.3.5. Elemental Analysis (EA)

EA was performed in order to quantify the sulfate groups (S%) of hydrolyzed BCNCs using a Euro EA3000 Elemental Analyzer from Eurovector (Pavia, Italy). Samples were measured using the SCAB.PE.29.PR.10.02 method in the solid state, in which the sample is combusted in presence of oxygen, and the resultant gaseous products are analyzed through gas chromatography (GC) equipped with a thermal conductivity detector. The signals were analyzed using Callidus^®^ software, which automatically provided the sample elemental composition report.

### 2.4. GPPE Extraction and Characterization

#### 2.4.1. Ultrasound-Assisted Polyphenol Extraction

Polyphenolic extract was obtained through ultrasound-assisted extraction from 122 mg of previously freeze-dried and ground GP with 3 mL of MeOH:H_2_O:HAc (30:69:1, *v*/*v*/*v*) solvent mixture and ascorbic acid (2 g/L) as antioxidant [33]. The mixture was first stirred in a vortex for 1 min and the extraction was performed in an ultrasonic bath for 15 min. Then, the hydroalcoholic solution was centrifuged for 4 min at 4000 rpm and 10 °C and the supernatant was dried in a rotational evaporator (Heidolph Hei-Vap Core, Schwabach, Germany) at 100 mmHg and 50 °C. Finally, the extract was freeze-dried to remove residual water and stored in a desiccator in the dark until use. 

The extraction of polyphenols for the oxidative and physical stability measurements followed a similar procedure, but, in this case, ascorbic acid was not added in order to not interfere with the intrinsic antioxidant activity of the GPPE, and the ultrasound treatment was extended to 20 min at 50 °C [34]. 

#### 2.4.2. Ultra-High-Performance Liquid Chromatography (UHPLC)

The identification of the polyphenolic profile was achieved using UHPLC of the supernatant of the centrifuged GPPE. The UHPLC was performed with an ACQUITY UPLC^TM^ system from Waters (Milford, MA, USA), equipped with a binary solvent delivery pump, an autosampler, a column compartment and a PDA detector. A reverse-phase column (Acquity UPLC BEH C18 2.1 × 100 mm, 1.7 µm) and a precolumn (Acquity UPLC BEH C18 1.7 µm VanGuard^TM^) from Waters (Milford, MA, USA) were used at 40 °C for the separation. The flow rate was 350 µL/min and the injection volume was 2.0 µL. Mobile phases consisted of 0.1% acetic acid in water (A) and 0.1% acetic acid in acetonitrile (B). The gradient conditions for the separation were as follows: 0–1.6 min, 2% B isocratic; 1.6–2.11 min, linear gradient from 2% to 8% B; 2.11–8.80 min, 8% B isocratic; 8.80–9.80 min, linear gradient from 8% to 10% B; 9.80–17.00 min, 10 % B isocratic; 17.00–22.00 min, linear gradient from 10 to 20% B; 22.00–23.40 min, linear gradient from 20% to 23% B; 23.40–54.20 min, linear gradient from 23% to 60% B; 54.20–55.20 min, linear gradient from 60% to 100% B, finally followed by washing and conditioning of the column. The temperature of the samples was maintained at 4 °C during the analysis. The wavelength range of the PDA detector was 210–500 nm (20 Hz, 1.2 nm resolution). Hydroxybenzoic acids were monitored at 254 nm, flavanols at 280 nm, hydroxycinnamic acids at 320 nm and flavonols and dihydroflavonols at 370 nm. Once the chromatographic peaks corresponding to polyphenols were assigned, their identity was studied busing mass spectrometry [33].

#### 2.4.3. Mass Spectrometry (MS)

MS data acquisitions were performed on a SYNAPT G2 HDMS with a quadrupole time-of-flight (QTOF) configuration (Waters, Milford, MA, USA) equipped with an electro-spray ionization (ESI) source operating in positive and negative modes. The capillary voltage was set to 1.0 kV for both ESI+ and ESI-. Nitrogen was used as the desolvation and cone gas at flow rates of 1000 L/h and 10 L/h, respectively. The source temperature was 120 °C, and the desolvation temperature was 400 °C. The two acquisition modes used were MS^E^ and MS/MS. Data acquisition took place across the 50–1200 *m*/*z* mass range in resolution mode (FWHM ≈ 20.000), and the scan time was 0.1 s. The collision energy for MS^E^ was 4 V in the trap cell and 4 V in the transfer cell for Function 1, and a collision ramp of 10 to 40 V in the trap cell and 4 V in the transfer cell for Function 2. The collision energy for MS/MS was a collision ramp of 10 to 40 V in the trap cell and 4 V in the transfer cell.

### 2.5. BCNC-GPPE Complex Preparation and Characterization

#### 2.5.1. BCNC-GPPE Complex Preparation 

Firstly, a homogenous dispersion of BCNCs was prepared through ultrasonic treatment at 750 W, 20 kHz and 30% amplitude of the ultrasonic device (Vibracell 75041, Bioblock scientific, Illkirch-Graffenstaden, France). Then, once the BCNCs were rehydrated and well dispersed, GPPE aqueous solution was gradually added to the BCNC dispersion. The mixture was kept under gentle agitation overnight, and the final dispersions gave BCNC:GPPE mass ratios of 1:1, 2.5:1 and 5:1 for a fixed GPPE concentration of 0.25 mg/mL.

#### 2.5.2. Dynamic Light Scattering (DLS) 

The stability of both the BCNCs and BCNC-GPPE dispersions was analyzed using DLS, measuring the Z potential using a ZetaSizer Nano Series ZEN3600 (Malvern Instruments, Malvern, UK). Surface charge was measured at different pHs, and values were taken in triplicate in specific cells from Malvern Instruments (DTS1070, Malvern, UK). Samples were prepared by diluting the dispersions with ultrapure water. 

#### 2.5.3. UV–Visible Spectrophotometry (UV-vis)—Antioxidant Activity Assay

The oxidative stability of the free GPPE and BCNC-GPPE dispersions was measured over time according to the DPPH free radical method, following the procedure described by Ventura-Aguilar et al., with some modifications [35]. Trolox was used for the calibration curve. The DPPH method was used in the free radical DPPH absorbance reduction when mixed with a substance with antioxidant activity, with the formation of DPPH-H and free radical of the antioxidant species. In particular, the loss of antioxidant capacity of the samples exposed to light, in the dark and in the dark at 4 °C was monitored for 40 days, selecting days 1, 9, 20 and 40 as time points. In order to observe the effect of BCNCs on the antioxidant capacity of the GPPE, BCNC-GPPE ratios of 1:1, 2.5:1 and 5:1 were studied. 

Shortly, 250 mL of either GPPE 2.5 % *w*/*v* solution or BCNC-GPPE dispersion was added to 2 mL of DPPH solution (6.25 × 10^−5^ M) in methanol, and left to stand in the dark for 30 min at room temperature. After this period, the absorbance was measured at 517 nm in 1 mL quartz cuvettes using UV-vis equipment. Measurements were conducted in triplicate, and the results are expressed in mg Trolox equivalent (TE) per g of dry extract (mg TE/g dry GP). With the purpose of comparing the degradation of the BCNC-GPPE complex and free GPPE, a first-order reaction kinetic model was used. Degradation rate constants (λ) and half-lives (t_1/2_) were calculated according to Equations (3) and (4), respectively:

(3) $$\ln\frac{C}{C_{0}}\;=\;-\;\lambda \;t$$

(4) $$t_{1/2}\;=\;\frac{\ln2}{\lambda }$$

where *C*_0_ is the initial mg TE/g GPPE and *C_t_* is the mg TE/g GPPE at time t.

Additionally, ABTS radical scavenging and total phenolic content (TPC) assays were performed to further characterize the antioxidant activity of GP. The ABTS radical cation, which is a blue chromophore, was generated through the reaction of ABTS with a 0.6 mM potassium persulfate solution (1:1) in the dark for 16 h at room temperature [36]. Trolox was employed as a standard in the assay, and a calibration curve was established in the concentration range of 10–50 mg Trolox/L. A mixture of 250 mL of GPPE or standard diluted with methanol and 2 mL of the ABTS^·+^ solution was prepared, and the absorbance of the samples was measured at 734 nm using a UV-vis spectrophotometer after 40 min. The experiment was conducted in triplicate, and the results are reported as mg Trolox equivalent per gram of dry GP (mg TE/g dry GP). 

The TPC method was modified from the procedure developed by Diñeiro et al. [37]. Gallic acid (GA) was utilized as a standard in the assay, and the calibration curve was created in a concentration range of 5–400 mg GA/L. A mixture of 0.1 mL of GPPE or standard diluted with methanol, 2 mL of a 20% Na_2_CO_3_ solution, 5 mL of H_2_O and 0.5 mL of Folin–Ciocalteu reagent was prepared in a test tube. The absorbance was measured at 765 nm using a UV-vis spectrophotometer after 30 min at 40 °C, and the results were determined using the calibration curve. The findings are reported as mg of GA equivalent per g of dry GP (mg GA/g dry GP).

### 2.6. Pickering Emulsions

#### 2.6.1. Oxidative Stability

The oxidative stability of 10 mL oil-in-water emulsions prepared with 10% *w*/*w* olive oil and stabilized by the BCNC (2.5 mg/mL) and BCNC-GPPE complexes (2.5:1 mass ratio) was assessed by measuring the increase in primary oxidation products of a polyunsaturated acid [38]. Additionally, another emulsion was prepared with the commercial emulsifier Tween 20 (TW20) as a control. The emulsions were prepared through tip sonication using a 3 mm diameter titanium probe at 750 W, 20 kHz and 30% amplitude (Vibracell 75041, Bioblock scientific). Each sample was then subjected to 3 s ultrasound and 3 s standby cycles of 20 s. The formation of conjugated dienes (CD) was monitored for 4 days by analyzing their absorbance at 233 nm (Shimadzu UV–Vis-NIR 3600). The emulsions were subjected to the Schaal oven test and kept in the dark at 60 °C [39]. All samples were vortexed for 1 min every 24 h to maintain the emulsion’s physical integrity. At different time points, 100 µL of each sample was diluted in 10 mL ethanol and analyzed in the UV spectrophotometer. The oxidative experiments were carried out in triplicate.

#### 2.6.2. Physical Stability 

Hexadecane-in-water Pickering emulsions were prepared in a 30:70 ratio, with the same ultrasonic device, to study the physical stability of the emulsions [40]. The aqueous phase was prepared with concentrations of 1, 2.5 and 5 mg/mL of previously dispersed BCNCs and BCNC-GPPE complexes. The emulsion stability was tested by subjecting the samples to 4000× *g* for 5 min [11]. The result was a milky white Pickering emulsion with a creamy phase of emulsified hexadecane close to the surface. After 24 h, the creamy phase started to condense on the surface, and its height was measured with a digital caliper. Simultaneously, a transparent aqueous phase emerged at the bottom of the emulsions. The emulsion ratio (ER) was calculated with the following equation [41]:

(5) $$ER\;(\%)\;=\;\frac{H_{c}}{H_{e}}\;\cdot \;100$$

where *H_c_* is the height of the creamy phase and *H_e_* the total height of the emulsion. 

In order to observe the possible coalescence of the different phases over time, 50 µL of the emulsion was dissolved in 1 mL of water after 7 days. A drop of this solution was then deposited onto a glass slide and observed in an optical microscope (Nikon Eclipse 80i) at a magnification of 10×. Subsequently, Image J software was used to analyze the emulsion droplet size of the optical micrographs. Finally, the diameter was calculated as the volume mean diameter D [4,3], employing the following equation:

(6) $$D\left[4,3\right]\;=\;\frac{\sum n_{i}d^{4}}{\sum n_{i}d^{3}}$$

where *n*_i_*d* is the diameter of the oil drops in the Pickering emulsion. 

#### 2.6.3. Rheological Properties 

Rheological measurements were performed for hexadecane-in-water Pickering emulsions stabilized by the BCNC and BCNC-GPPE complex using a TA Instruments ARES G2 rheometer. Dynamic oscillatory strain sweep and frequency sweep tests as well as flow tests were performed at 25 °C using cone-plate geometry (50 mm diameter). The linear viscoelastic region (LVR) was determined by monitoring both the storage modulus (G’) and loss modulus (G’’) in a strain sweep test at 1 Hz. After that, G’ and G’’ were analyzed versus frequency from 0.1 to 100 Hz at a fixed strain within the LVR region. Flow test measurements were performed, in which the evolution of the viscosity was measured by varying the shear rate from 0.01 to 1000 s^−1^. All experiments were performed in triplicate.

### 2.7. Statistical Analysis

In this study, R version 4.1.2 (R Core Team, 2021) was used to perform statistical analyses, including a two-sample t-test to compare means between groups. All experiments were conducted at least in triplicate, and differences were considered to be statistically significant when *p* < 0.05.

## 3. Results and Discussion

### 3.1. BCNC Production and Characterization

#### 3.1.1. BCNC Particle Size Distribution

**Figure 1:**
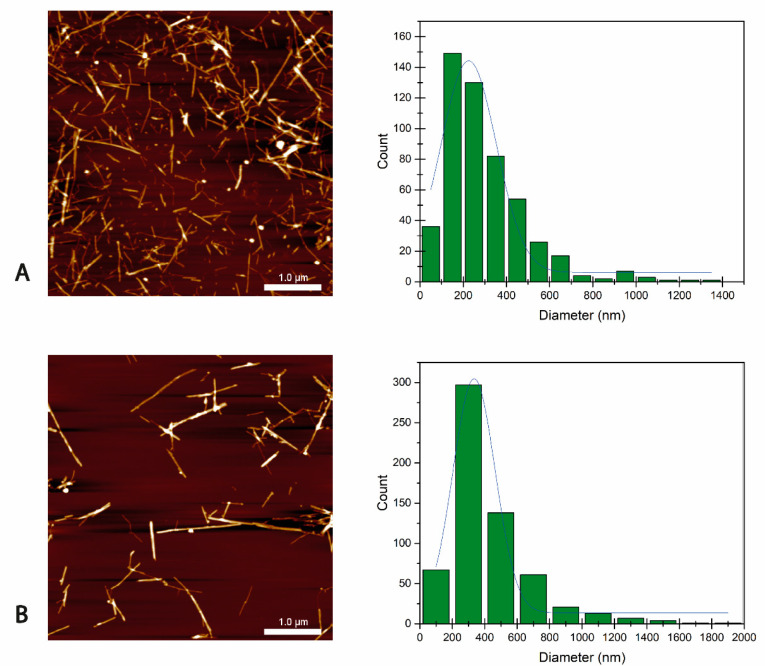
AFM images and size distribution of BCNC EH (**A**) and BCNC AH (**B**), respectively.

The morphology (size and shape) of BCNCs is a key factor in the formation of Pickering emulsions [41,42]. The rod-like BCNCs were successfully obtained through both EH and AH, as can be observed in the AFM images in Figure 1A,B, respectively. According to the images, their sizes varied up to 1.5 µm in length and between 5 and 30 nm in width, confirming their high aspect ratio (Figure 1A,B). As seen, likely due to enzyme type diversity, the obtained BCNCs showed large polydispersity in size, which was slightly higher in the case of EH. Thus, smaller nanocrystals could be found together with some nanofibers exceeding 1 µm. This broad size distribution could favor the final applicability of the BCNCs, since, unlike the inorganic spherical particles commonly used for Pickering emulsions, the combination of large aspect ratio rod-shaped particles and nanofibers could form bridge structures that contribute to stabilizing the emulsion by creating entangled networks [11,42]. 

Regarding the hydrolysis, the obtained BCNC yield of the EH procedure calculated relative to the initial dry BC was close to 35%. This value was in line with those reported in the literature for EH [31,43], indicating that despite being lower than those of the traditional AH process [12,44,45], the selective and sustainable EH method was successfully carried out. It should be pointed out that BC membranes were enzymatically hydrolyzed in the wet state after the defibrillation of the membrane, thus increasing the surface area and facilitating the enzyme activity. However, increasing the usually low EH yield value is one of the topics of great interest in order to completely substitute the unsustainable AH process.

#### 3.1.2. BCNC Characterization

**Figure 2:**
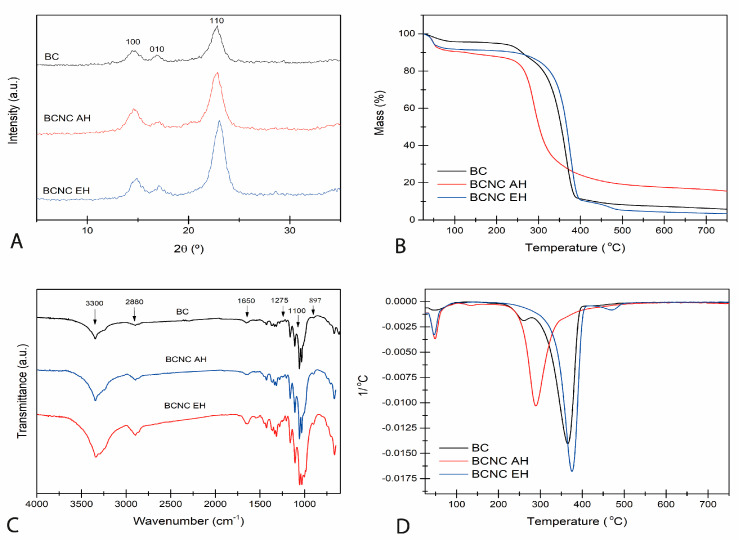
Physicochemical characterization and thermal degradation profile of BC, BCNC EH and BCNC AH samples. XRD (**A**), TGA (**B**), FTIR (**C**) and dTG (**D**).

The crystalline structure of both the BC membrane and BCNCs was analyzed using XRD (Figure 2A). The patterns corresponded to the typical cellulose I allomorph, with three main peaks located at 2θ = 14.5°, 16.8° and 22.7° related to the crystallographic planes (100), (010) and (110), respectively. The CIs calculated using the Segal equation (Equation (2)) were 77% for the BC membrane, 87% for BCNC EH and 86% for BCNC AH, in close agreement with the CI values found in literature [12,46]. These results demonstrated that EH effectively removed amorphous regions of the original BC, and high crystalline nanocrystals could be obtained. It is worth noting that even though the wet state of the BC resulted in mild acidic conditions [40], the risk of the commonly reported allomorphic transformation from cellulose I to cellulose II of AH [47] further recommends the EH alternative. Furthermore, some authors have suggested that the allomorph cellulose I, which is more hydrophobic, would be preferable to cellulose II for stabilizing Pickering emulsions and reinforcing polymers [48,49]. 

The obtained FTIR spectra of the BC membrane and different BCNCs are shown in Figure 2C. In agreement with the XRD spectra, the cellulose I allomorph was identified in all samples through the characteristic absorption bands at 1427, 1280 and 897 cm^−1^ [50]. The bands located around 3300 cm^−1^ were attributed to the stretching vibration of O-H linkages. The absorption bands at 2900–2880 cm^−1^ and 1460–1250 cm^−1^ corresponded to the CH and CH_2_ stretching and bending vibrations, respectively. Similarly, vibrations of the C-O-C bond of the glycosidic bridges were identified by the peaks at 1170–1050 cm^−1^. The broad band at 897 cm^−1^ is characteristic of β-linked glucose-based polymers. Finally, the band at around 1650 cm^−1^ was associated with the absorbed water. In agreement with the XRD results, the increase in crystallinity in BCNC samples could be verified by the sharpening of the broad band between 3400 and 3000 cm^−1^ corresponding to hydrogen-bonded hydroxyl groups [51].

The thermal stability of BC and BCNC was analyzed using TGA underrun a nitrogen atmosphere. The obtained thermograms and the corresponding derivative curves (dTG) are displayed in Figure 2B,D. As shown in Figure 2B, the curves followed the expected cellulose decomposition profile, in which three major events were distinguished. The first event, near 100 °C, corresponded to the evaporation of surface adsorbed humidity and low-molecular weight compounds. Then, cellulose depolymerization, dehydration and the decomposition of glycosidic bonds took place in a second event between 250 and 450 °C. In the case of the BC membrane sample, a small decrease and a peak at 250 °C can be observed in TGA and dTG in Figure 2B,D, respectively. This drop was associated with possible residual organic debris from the biosynthesis process. As can be observed, BCNCs obtained via the enzymatic route showed higher thermal stability than those arising from the acidic route, and the degradation was moderately later than that of native BC [12,30]. The esterification of cellulosic hydroxyl groups and subsequent sulfate group formation of the AH might decrease thermal degradation activation energy of cellulose [52] and, thus, accelerate the thermal decomposition of BCNCs. Finally, the ash percentage calculated at 750 °C was lower in the BC and BCNC EH samples, also likely related to sulfate groups that acted as dehydration catalysts [51]. 

### 3.2. GPPE Extraction and Characterization

The identification of the polyphenolic compounds in the GPPE was performed using UHPLC coupled to MS. These types of polyphenolic extracts stand out for having a wide variety of complex phenolic molecules [53]. Moreover, depending on the grape variety, ripening stage or the growing region, the extract composition may change. In particular, white GP has an extraordinarily higher polyphenol concentration than that found in red grapes [54], since white wine is not usually subjected to prior maceration processes and the pomace is discarded directly after grape pressing [55]. According to the UHPLC-MS analysis (Figure S1 and Table S1), the obtained extract presented polyphenols belonging mainly to flavanols, flavonols and hydroxycinnamic and hydroxybenzoic acids. The most numerous group corresponded to that of the flavonols, where procyanidin and procyanidin trimer were identified. In addition, signals attributed to catechin, epicatechin and procyanidin gallate could be detected. Among the flavanols, different glycosides of quercetin, kaempferol and isorhamnetin were mainly identified. Hydroxycinnamic acids such as as p-coumaroyl hexose were also found, likely coming from grape skin. Finally, gallic acid and galloyl derivatives were detected within the group of hydroxybenzoic acids. These results are in agreement with those published in the literature for similar white GPs [26,54]. 

Due to the variety of mechanisms of action of polyphenolic extracts, DPPH, ABTS, and TPC assay methods for measuring antioxidant activity were used [26]. The antioxidant activity values exhibited by GPPE were 174.44 ± 15.49 (mg TE/g dry GP), 281.43 ± 86.12 (mg TE/g dry GP) and 195.52 ± 56.15 (mg GA/g dry GP), respectively. These findings are consistent with the wide variety of polyphenolic compounds observed in the UHPLC-MS analysis. The antioxidant activity results per dry matter obtained are in the same order of magnitude and even slightly higher than those found in the literature for residues from the wine industry, thus demonstrating the excellent antioxidant properties of white grape pomace [24,39,40]. 

Ultrasound and hydroalcoholic solvent methods have separately proven to be effective even at low temperatures [27,34,56]. Indeed, higher temperatures help to improve the extraction yield, but it has been demonstrated that temperatures above 60 °C can irreversibly degrade the polyphenols [25,57]. Likewise, shock waves of ultrasound can damage the plant cell wall, facilitating the extraction of desired compounds into the extraction solvent. Nevertheless, despite the advantages of using ultrasound-assisted extractions, it has rarely been used for the extraction of polyphenols from grapes, and, in fact, a standardized protocol for this type of extraction does not yet exist [58]. In view of the results, the combination of ultrasound and hydroalcoholic solvents at mild temperatures appears to be a promising strategy that preserves the intrinsic bioactivity of polyphenols [5,25,27,38]. 

### 3.3. BCNC-GPPE Complex Characterization

#### 3.3.1. Z Potential

**Figure 3:**
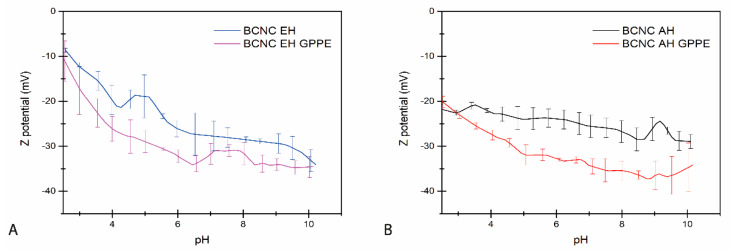
Z potential value evolution of BCNC EH and BCNC EH-GPPE (**A**) and BCNC AH and BCNC AH-GPPE (**B**) dispersions.

BCNCs obtained both from the EH and AH treatments were combined with the GPPE for the preparation of aqueous dispersions that gave BCNC-GPPE ratios of 2.5:1 (*w*/*w*). The Z potential values of the dispersions were measured as indications of the surface charge and the stability of dispersions of bare BCNCs and the BCNC-GPPE complex. Figure 3A,B shows the Z potential of BCNC EH and BCNC AH dispersions at different pHs, respectively. As can be observed, the BCNCs showed negative Z potentials in all cases that gradually increased with the pH, in agreement with published reports [14,49]. However, BCNC AH showed slightly more negative Z potential values than BCNC EH at acidic pHs. The decrease in surface charge could also be attributed to the sulfate groups produced during the AH [6]. The decreasing Z potential indicated that above pH 6, BCNCs had sufficient negative charge and, thus, repulsive forces to produce stable colloids (<−25 mV) [6,12,59,60]. However, the Z potential of the BCNC-GPPE complex dispersion was lower in both cases, indicating more negative surface charge and higher colloidal stability in the complexes, even at pH 4. Polyphenolic extracts are rich in both hydroxyl and carboxylic acid groups and, therefore, GPPE contributes to increasing the negative charge of the complexes once deprotonated, thus improving colloidal properties [61]. Therefore, the combination of GPPE and BCNC adjusted the surface hydrophobicity of nanocrystals and increased repulsive forces, which resulted in particular interest for BNCN EH at lower pH levels [4,62]. In this way, the complexes promoted an easier, more economical and environmentally friendly approach of tuning the hydrophobicity and interfacial tension of the nanocrystals [63]. 

#### 3.3.2. Antioxidant Activity Assays 

The stability of the antioxidant activity of both the GPPE and BCNC-GPPE complex at different concentrations was measured using the DPPH method. For this purpose, samples were subjected to different temperature and visible light conditions for 40 days. It should be noted that DPPH was chosen as the analytical method for the degradation study, and the antioxidant capacity of some of the previously mentioned polyphenols may not have been fully represented. Table 1 shows the half-life (t_1/2_) of the antioxidant activity loss for each sample, calculated according to Equations (3) and (4). 

With respect to the storage conditions, the collected data showed that light exposure and ambient temperature accelerated the degradation of the polyphenols. As observed, the formation of BCNC-GPPE complexes notably delayed the degradation and the loss of antioxidant activity of the GPPE in all storage conditions, demonstrating the usefulness of polyphenol encapsulation [25,64]. The higher the used BCNC:GPPE ratio, the longer the antioxidant capacity lasted over time, with the best obtained ratios of 5:1, enhancing the protective activity even in the more aggressive storage conditions. In this regard, it is worth mentioning that BCNC AH presented a longer protective effect than BCNC EH, likely due to their slightly better colloidal stability, which might protect the most-exposed polyphenolic hydroxyl groups [65]. These findings demonstrated that GPPE can form stable and active complexes with BCNCs with prolonged antioxidant capacity over time suitable to be used as a potential active stabilizer for the cosmetics, food and pharmaceutical industries. Finally, although some authors had previously reported encapsulation of plant-derived polyphenols by cellulose or its derivatives [22,66,67], in this work, prolonged polyphenol degradation protection was achieved through an integral agricultural waste valorization strategy. 

### 3.4. Pickering Emulsion Characterization

#### 3.4.1. Oxidative Stability 

**Figure 4:**
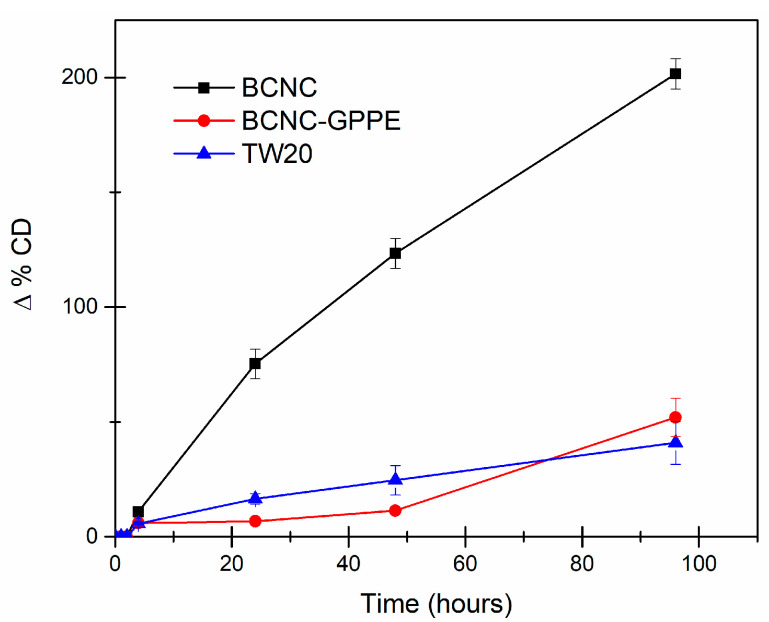
BCNC-, BCNC-GPPE complex- and TW20-stabilized olive oil-in-water emulsion’s oxidative stability measured through CD increase.

The oxidative stability of lipids is a key parameter to validate the stability of an O/W emulsion since they are responsible for off-flavors as well as of the loss of nutritional attributes, especially in the cosmetic and food industries [18,68,69]. In order to assess the active protection capacity of the prepared BCNC and the BCNC-GPPE complex in Pickering emulsions, three olive oil-in-water emulsions were prepared using TW20 (control), BCNC and the BCNC-GPPE complex, respectively. Their oxidative stability was studied by monitoring the evolution of the UV absorbance at 233 nm, associated with the formation of primary lipid oxidation products as CD (Figure 4). Considering the low percentage of CD formation in the TW20-stabilized control emulsion, in absence of an additional antioxidant agent, the complete coverage of the oil–water interface by the surfactant could be concluded. In case of Pickering emulsions, effective bridging structures and the tight packing of solid particles at the interface may also prevent direct contact with oxygen or free radicals and, thus, improve oxidative stability. Li et al. attributed this protective capacity to BC nanofibers in their curcumin-loaded Pickering emulsions [21]. As can be clearly observed in Figure 4, the BCNC-stabilized olive oil Pickering emulsion showed substantially higher CD formation than that of the control. This finding indicated that the BCNCs did not achieve the required degree of interface coverage to effectively protect against the oxidation of the olive oil. In contrast, the protective activity of the BCNC-GPPE complex was successfully assessed, demonstrating that it contributed to inhibiting the oxidative reactions of the olive oil lipids, even after four days of the Schaal oven test. Indeed, the BCNC-GPPE Pickering emulsion presented similar protective activity against oxidation to that of the control, and no statistically significant differences were observed at the end of the test. Furthermore, in the first 48 h, the active protection of the BCNC-GPPE complex seemed to be higher. These results are consistent with those published by Pazos et al., where flavanol oligomers extracted from grapes showed a potent antioxidant activity fir oil-in-water emulsions [70]. Similarly, Yi et al. observed that the addition of anthocyanins improved the physical stability and prevented lipid oxidation of whey protein-stabilized emulsions [24]. Certainly, the unique structure of polyphenols allowed them to scavenge free radicals by donating the phenolic hydroxyl proton [71,72]. In this context, GPPE could play a dual role, both scavenging radicals and contributing to the complete coverage of the oil–water interface of the emulsion [38,39,73,74].

#### 3.4.2. Physical Stability 

**Figure 5:**
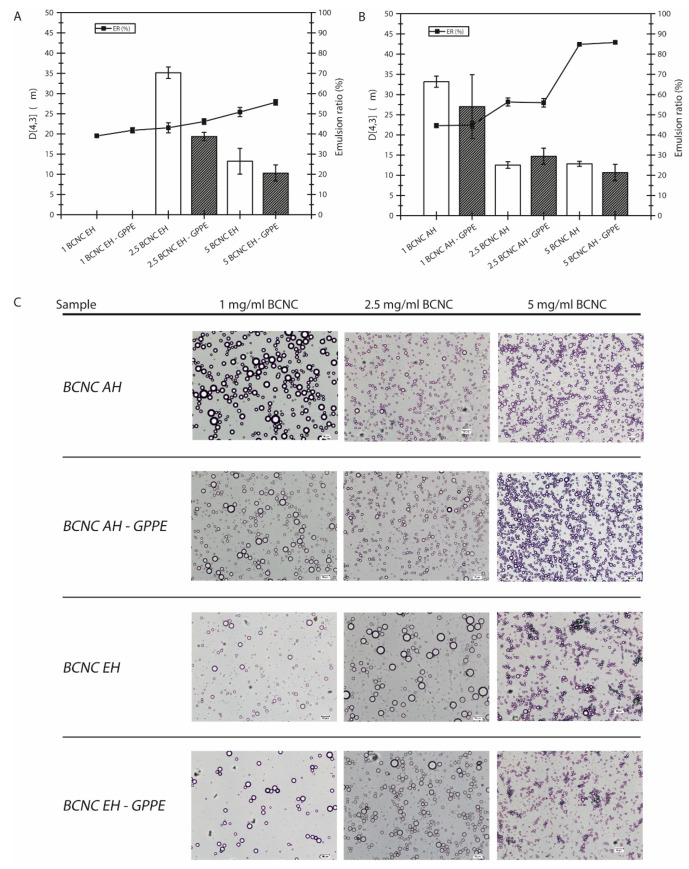
ER and emulsion drop D [4,3] of hexadecane-in-water Pickering emulsions stabilized by neat BCNC AH and BCNC AH-GPPE complex (**A**), and neat BCNC EH and BCNC EH-GPPE complex (**B**). Optical microscope images of hexadecane-in-water Pickering emulsions stabilized by different concentrations of BCNC AH, BCNC AH-GPPE complex, BCNC EH and BCNC EH-GPPE complex dispersions. GPPE concentration remained constant against increasing concentrations of BCNCs (**C**).

The capacity of the BCNCs to form stable Pickering emulsions was studied by preparing hexadecane-in-water emulsions with different concentrations of neat BCNC EH and BCNC AH, as well as with their corresponding BCNC-GPPE complexes. Different GPPE concentrations were also tested independently to evaluate its emulsification capacity, but in those cases, the emulsification could not be achieved, indicating that the GPPE did not present emulsification capacity by itself. The emulsion stability was analyzed by calculating both the ER and the D [4,3] values of BCNC EH- and BCNC AH-stabilized emulsions, as displayed in Figure 5A,B, respectively. In general, an emulsion is considered stable when both particle size and particle size distribution remain constant over an extended period of time. Physical disturbances such as centrifugation, filtration or agitation could accelerate the coalescence and phase separation of the emulsion. In this case, centrifugation was used to induce emulsion packing and accelerate the creaming phenomenon [12,40]. The higher the ER value, the better the emulsion stability and the resistance against centrifugation.

According to Figure 5, the ER value increased progressively with the concentration of BCNCs, and uniform white emulsions were obtained above 2.5 mg/mL [10,12,75]. The statistical analysis revealed that the differences were significant at each increase in BCNC concentration compared to the previous one. Nevertheless, in the case of 1 mg/mL BCNC, with lower emulsification capacity, hexadecane droplets began to coalesce and rise to the surface after centrifugation, creating a thin hexadecane layer. The emulsified phase of these samples was characterized by a small number of huge droplets in suspension when observed with the optical microscope. This behavior usually occurs when the amount of stabilizing particles is not sufficient to emulsify the entire discontinuous phase [40,76]. 

Likewise, the droplet size results were consistent with those of ER, and each increase in BCNC concentration showed statistically significant differences in the reduction in D [4,3] in all samples (Figure 5A,B). In other words, the higher the BCNC concentration, the higher the number of droplets and the smaller the droplet diameter, as can be confirmed in the images displayed in Figure 5C [12,76,77]. Thus, it seemed that the decrease in the drop size as well as their spherical shape were favored by BCNCs, being effectively placed at the oil–water interface and preventing the droplets from coalescence. The ER values obtained for BCNC AH were higher than those of BCNC EH. Considering the slightly acidic pH of the prepared emulsions, near 4.5, the better performance of BCNC AH could be related to their better colloidal properties. This difference was more reduced at higher concentrations of BCNC, where steric hindrance and electrostatic repulsion are more dominant between BCNC-coated droplets [60]. However, the formation of the BCNC-GPPE complex had a positive influence on the ER values and droplet size in all BCNC EH emulsions, while this effect was not appreciable in the case of BCNC AH. In fact, this decreasing trend for D [4,3] showed statistically significant differences in the case of the 2.5BCNC EH-GPPE sample (Figure 5A). Polyphenolic extracts possess excellent amphiphilic properties due to their abundance in hydrophobic aromatic nuclei and hydrophilic groups that might contribute to the physical stability [17,18,73,78]. This feature of polyphenols was exploited by Hu et al. by chemically functionalizing CNCs to improve their colloidal properties [16]. Nevertheless, the results in the present work suggested a synergistic effect arising from the formation of the BCNC EH-GPPE complex, which led to improved emulsion stability without additional chemical treatments of BCNCs. This improvement coincided with the decrease in Z potential and the delay in the appearance of the undesirable CD in the case of the BCNC-GPPE complex. Moreover, physical stability enhancement may also positively influence the oxidative stability, as was demonstrated with BCNC-GPPE [79]. The results demonstrate that Pickering emulsions with prolonged antioxidant properties can be obtained through a green and fully sustainable valorization strategy of an agricultural residue such as GP. 

#### 3.4.3. Rheological Behavior 

**Figure 6:**
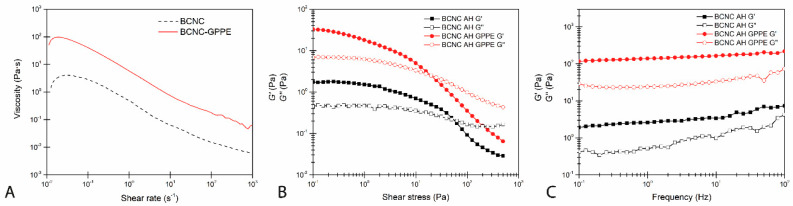
Rotatory and oscillatory rheological measurements of hexadecane-in-water BCNC and BCNC-GPPE Pickering emulsions. (**A**) Flow test, (**B**) dynamic oscillatory strain test and (**C**) frequency sweep tests. Storage modulus (filled symbols) and loss modulus (empty symbols).

In order to further confirm the improvement in the physical stability, the rheological behavior of hexadecane-in-water Pickering emulsions stabilized by BCNC and the BCNC-GPPE complex was studied. As depicted in Figure 6, both the BCNC and BCNC-GPPE emulsions presented a pseudoplastic character and showed the classic shear thinning behavior in which a decrease in their viscosities with the increasing shear rate was observed [79,80]. The emulsions stabilized by the BCNC-GPPE complex presented higher apparent viscosity in the whole range, thus suggesting that the addition of GPPE contributed to creating a stronger structural network. 

Dynamic oscillatory strain and frequency sweep test results are displayed in Figure 6B,C, respectively. Both the BCNC- and BCNC-GPPE complex-stabilized emulsions presented gel-like rheological behavior, with G’ above G’’. However, emulsions stabilized with the BCNC-GPPE complex showed higher moduli values, indicating stronger network structure formation in this case. At the same time, emulsions presented gel-like behavior in the LVR, where both moduli remained constant in the whole frequency range. Again, the BCNC-GPPE emulsion obtained higher values in the studied range and, moreover, the gap between the two moduli remained even at the highest frequency values, whereas in the case of BCNC-stabilized emulsions, the gel-like network character weakening was noticed since the G’’ value approached to the G’. Thus, it could be deduced that GPPE improved the structural strength as well as the physical stability of the emulsion [21]. 

## 4. Conclusions

The results of the present study demonstrated a promising approach towards the comprehensive valorization of GP agro-industrial waste. On the one hand, its nutrients were utilized for the biosynthesis of BC, which, in turn, could be enzymatically treated to obtain high-purity BCNCs. On the other hand, the wide variety of polyphenols in GP were effectively extracted by a novel combination of ultrasound and a hydroalcoholic solvent. Thus, the typical polyphenolic compounds of white GP were identified, and their bioactive properties were confirmed through antioxidant capacity assays. The creation of the BCNC-GPPE complex had a protective effect on the polyphenols, prolonging the half-life of their antioxidant capacity. Additionally, the polyphenols acted as modulators of the surface charge of the BCNCs, improving their colloidal properties. As a result, the complex showed an increased ability to stabilize Pickering emulsions with antioxidant activity. Furthermore, the use of more sustainable methodologies increased the added value of the product and improved its applicability in the cosmetic, nutraceutical, and pharmaceutical sectors. Similarly, the synergistic effect observed between BCNCs and GPPE could be further studied. In addition to food-grade Pickering emulsion formation, the enhanced lipophilic affinity opens up a range of promising applications for cellulose–polyphenolic extract complexes, such as the recovery of contaminating oils, modulation of lipid digestion or the development of antimicrobial biopolymers.

## Figures and Tables

**Table 1:** The half-lives of DPPH free radical scavenging activity were measured for bare GPPE and the BCNC AH-GPPE and BCBN EH-GPPE complexes under various light and temperature conditions.

BCNC:GPPE	Samples	*t_1/2_* (Days)	*r^2^*
0:1	light	43.5	0.96
	dark	64.8	0.97
	dark + 4 °C	97.9	0.97
1:1	AH light	46.8	0.99
	AH dark	45.9	0.98
	AH dark + 4 °C	121.6	0.80
	EH light	43.6	0.99
	EH dark	55.5	0.99
	EH dark + 4 °C	96.3	0.96
2.5:1	AH light	46.2	0.97
	AH dark	68.0	0.92
	AH dark + 4 °C	150.7	0.80
	EH light	42.3	0.95
	EH dark	67.3	0.80
	EH dark + 4 °C	106.6	0.86
5:1	AH light	41.0	0.90
	AH dark	111.8	0.93
	AH dark + 4 °C	256.7	0.90
	EH light	37.3	0.92
	EH dark	68.6	0.78
	EH dark + 4 °C	130.8	0.78

## Data Availability

The data are contained within the article and Supplementary Materials.

## Supplementary Materials

The following supporting information can be downloaded at: https://www.mdpi.com/article/10.3390/antiox12051064/s1, Figure S1: Diode array detector (DAD) chromatogram of GPPE at 254 nm, 280 nm, 320 nm and 370 nm. Figure S2: Schematic representation of the entire experimental process carried out in this work. Table S1: Characterization of polyphenols from grape pomace extract determined through UHPLC-Q-TOF-MS/MS analysis.
