# Supplementary material for: Integral Valorization of Grape Pomace for Antioxidant Pickering Emulsions
Source: Antioxidants (Basel). 2023 May 8;12(5):1064. doi: 10.3390/antiox12051064 (PMC10215931; doi:10.3390/antiox12051064)
Supplement: Supplementary file 1 [file antioxidants-12-01064-s001.zip › Figure S2.pdf]

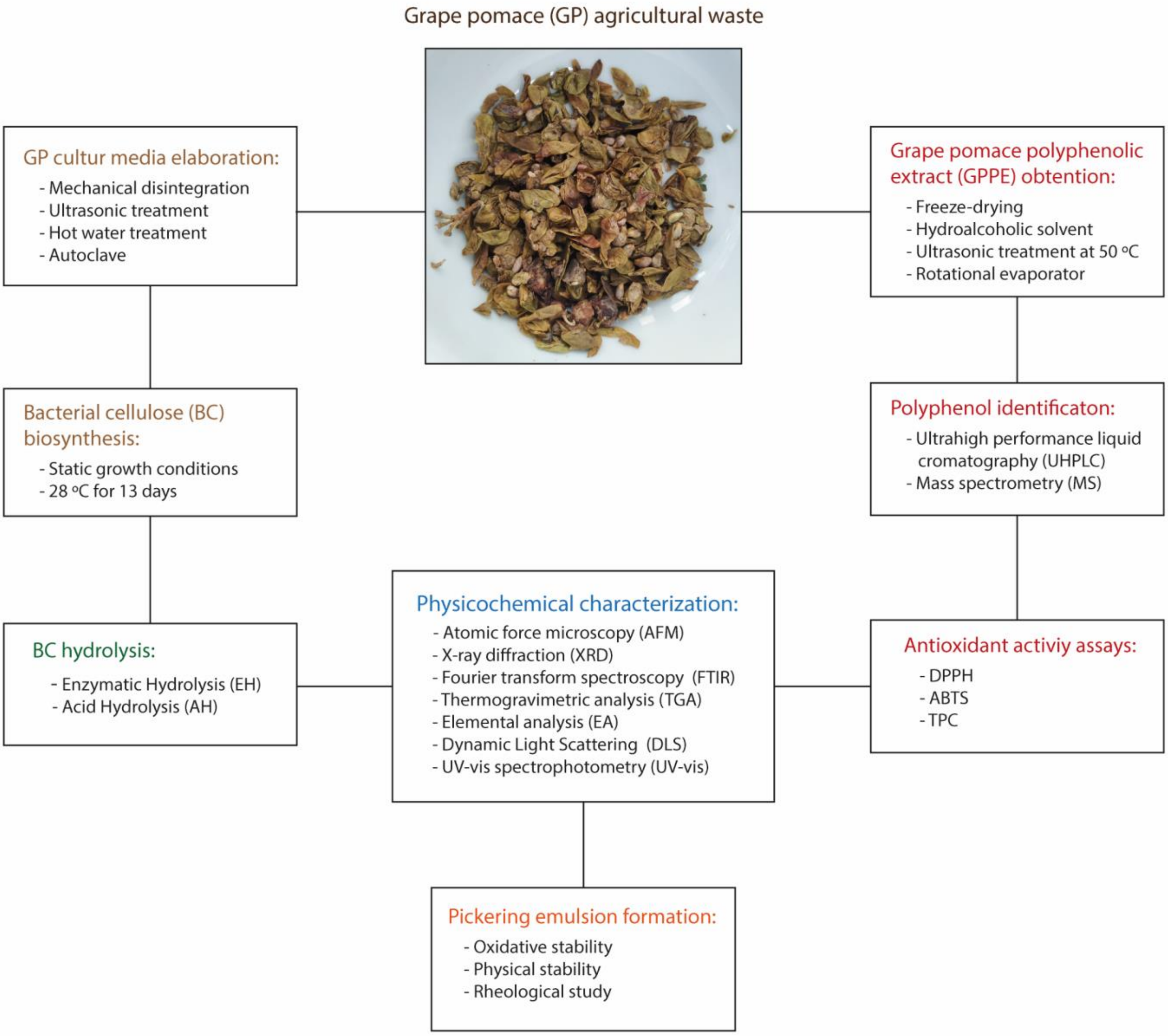

Figure S2. Schematic representation of the entire experimental process carried out in this work.
