# Supplementary material for: Integral Valorization of Grape Pomace for Antioxidant Pickering Emulsions
Source: Antioxidants (Basel). 2023 May 8;12(5):1064. doi: 10.3390/antiox12051064 (PMC10215931; doi:10.3390/antiox12051064)
Supplement: Supplementary file 1 [file antioxidants-12-01064-s001.zip › Supp material Julen Fig 1S and Table 1S.pdf]

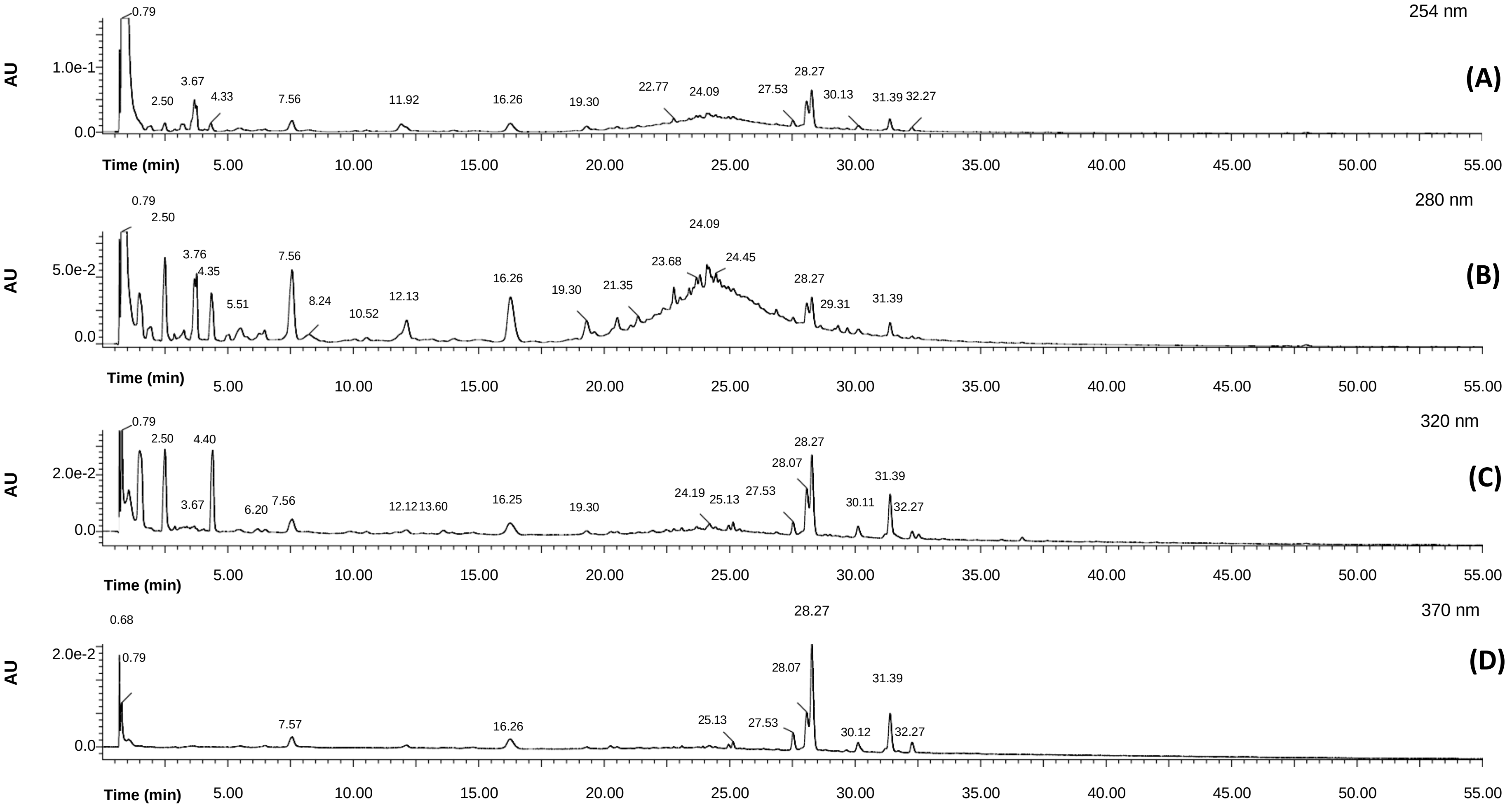

Figure S1. Diode array detector (DAD) chromatogram of GPPE at; 254 nm (A); 280 nm (B); 320 nm (C); and, at 370 nm (D).

Table S1. Characterization of polyphenols from grape marc extract determined by UHPLC-Q-TOF-MS/MS analysis. Abbreviations: *Cat* (Catechin), *Epi* (Epicatechin), *Gal* (Galic acid), *gal* (galactose, galactosil, galactoside), *glc* (glucose, glycosyl, glycoside), *glcr* (glucuronic acid, glucuronide), *hex* (hexose, hexosyl, hexoside), *Iso* (Isorhamnetin), *Kaem* (Kaempferol), *PB* (Procyanidin), *((Epi)Cat)) 3* (Procyanidin trimer), *Que* (Quercetin) and *pCoum* (p-Coumaric acid, p-coumaroyl).

				ESI(+)-Q-ToF			ESI(-)-Q-ToF		
**Comp .No**	**Tentative identification**	**LC tR (min)**	**DAD UV Bands (nm)**	**Exp. Acc. Mass $[M+H]^+$ Error (mDa)**	**Formula for the detected $[M+H]^+$**	**Adducts and Fragment ions of $[M+H]^+$**	**Exp. Acc. Mass $[M-H]^-$ Error (mDa)**	**Formula for the detected $[M-H]^-$**	**Adducts and Fragment ions of $[M-H]^-$**
Flavanols									
**1**	((Epi)cat)3 (1)	3.30	283	867.2136<br>-0.2	C45H39O18	715.1639 [U(1,3A)MD]+<br>579.1514 [MD]+<br>427.1038 [U(1,3A)D]+<br>409.0928 [M(1,3A-H2O)D]+<br>291.0873 [U]+<br>289.0716 [D]+<br>247.0620 [U-C2H2O]+<br>139.0394 [D(1,3A)]+<br>127.0396 [D(1,4A+2H)]+<br>123.0446 [D(1,2B)]+	865.1980<br>1.8	C45H37O18	713.1509 [U(1,3A)MD]-<br>577.1340 [MD]-<br>425.0869 [U(1,3A)D]+<br>407.0772 [M(1,3A-H2O)D]-<br>289.00705 [D]-<br>287.0551[U]-<br>245.0446 [U-C2H2O]+<br>125.0231 [D(1,4A+2H)]-
**2**	PBI	5.52	280	579.1508<br>0.5	C30H27O12	427.1032 [U(1,3A)D]+<br>409.0925 [U(1,3A-H2O)D]+<br>291.0872 [D]+<br>289.0707 [U]+<br>287.0553[U(1,3A)D(1,2A-H2O)]+<br>247.0606 [U-C2H2O]+<br>139.0393 [D(1,3A)]+	577.1351<br>0.5	C30H25O12	425.0874 [U(1,3A)D]-<br>407.0773 [U(1,3A-H2O)D]-<br>289.0714 [D]-<br>245.0815 [U-C2H2O]+<br>125.0239 [D(1,4A+2H)]-

						127.0393 [D(1,4A+2H)]+<br>123.0444 [D(1,2B)]+			
3	((Epi)cat)3 (2)	5.84	283	867.2133<br>-0.3	C45H39O18	715.1653[U(1,3A)MD]+<br>579.1497 [MD]+<br>427.1021 [U(1,3A)D]+<br>409.0907 [M(1,3A-H2O)D]+<br>291.0858 [D]+<br>289.0703 [U]+<br>247.0593 [U-C2H2O]+<br>139.0389 [D(1,3A)]+<br>127.0386 [D(1,4A+2H)]+<br>123.0438 [D(1,2B)]+	865.1988<br>0.8	C45H37O18	713.1503 [U(1,3A)MD]-<br>577.1327 [MD]-<br>425.0864 [U(1,3A)D]-<br>407.0763 [M(1,3A-H2O)D]-<br>289.0697 [D]-<br>287.0549 [U]-<br>125.0234 [D(1,4A+2H)]-
**4**	PBII	6.51	280	579.1496<br>-0.7	C30H27O12	427.1029 [U(1,3A)D]+<br>409.0921 [U(1,3-H2O)D]+<br>291.0865 [D]+<br>289.0704 [U]+<br>287.0557 [U(1,3A)D(1,2A-H2O)]+<br>247.0600 [U-C2H2O]+<br>139.0388 [D(1,3A)]+<br>127.0389 [U(1,4A+2H)D)]+<br>123.0441 [D(1,2B)]+	577.1358<br>1.2	C30H25O12	425.0867 [U(1,3A)D]-<br>407.0761 [U(1,3A-H2O)D]-<br>289.0707 [D]-<br>245.0810 [U-C2H2O]+<br>125.0234 [D(1,4A+2H)]-
**5**	Cat	7.59	278	291.0873<br>0.4	C15H15O6	207.0659[Rup(A)]+<br>147.0447 [0,4B-2H2O]+<br>139.0394 [1,3A]+<br>123.0446 [1,2B]+	289.0717<br>0.5	C15H13O6	205.0502 [Rup(A)]-<br>137.0235 [1,3A]-<br>123.0235 [1,3B-CO]-<br>109.0284 [1,3A-CO]-

						119.0497 [0,4B-2H2O-CO]+			
**6**	((Epi)cat)3 (3)	7.69	283	867.2133<br>-0.3	C45H39O18	715.1628 [U(1,3A)MD]+<br>579.1509 [MD]+<br>427.1025 [U(1,3A)D]+<br>409.0922 [M(1,3A-H2O)D]+<br>291.0874[D]+<br>289.0713 [U]+<br>247.0609 [U-C2H2O]+<br>139.0396 [D(1,3A)]+<br>127.0392 [D(1,4A+2H)]+<br>123.0446 [D(1,2B)]+	865.1988<br>0.8	C45H37O18	713.1502 [U(1,3A)MD]-<br>577.1331 [MD]-<br>425.0871 [U(1,3A)D]-<br>407.0772 [M(1,3A-H2O)D]-<br>289.0711 [D]-<br>287.0551 [U]-<br>125.0237 [D(1,4A+2H)]-
**7**	PBIII	8.27	280	579.1500<br>-0.3	C30H27O12	427.1022 [U(1,3A)D]+<br>409.0917 [U(1,3-H2O)D]+<br>291.0861 [D]+<br>289.0710 [U]+<br>287.0551 [U(1,3A)D(1,2A-H2O)]+<br>247.0602 [U-C2H2O]+<br>139.0390 [D(1,3A)]+<br>127.0390[U(1,4A+2H)D]+<br>123.0439 [D(1,2B)]+	577.1349<br>0.3	C30H25O12	425.0872 [U(1,3A)D]-<br>407.0769 [U(1,3A-H2O)D]-<br>289.0718 [D]-<br>245.0810 [U-C2H2O]+<br>125.0244 [D(1,4A+2H)]-
**8**	((Epi)cat)3 (4)	8.95	283	867.2121<br>-1.5	C45H39O18	715.685 [U(1,3A)MD]+<br>579.1493 [MD]+<br>427.1021 [U(1,3A)D]+<br>409.0910 [M(1,3A-H2O)D]+<br>291.0851 [D]+	865.1988<br>0.8	C45H37O18	713.1511 [U(1,3A)MD]-<br>577.1340 [MD]-<br>425.0869 [U(1,3A)D]-<br>407.0763 [M(1,3A-H2O)D]-<br>289.0704 [D]-

						289.0712 [U]+			287.0561 [U]-
						247.0603 [U-C2H2O]+			245.0461 [U-C2H2O]+
						139.0396 [D(1,3A)]+			125.0229 [D(1,4A+2H)]-
						127.0391 [D(1,4A+2H)]+			
						123.0446 [D(1,2B)]+			
**9**	((Epi)cat)3 (5)	9.80	283	867.2099	C45H39O18	715.1680 [U(1,3A)MD]+	865.1988	C45H37O18	713.1501 [U(1,3A)MD]-
				-3.7		579.1483 [MD]+	0.8		577.1353 [MD]-
						427.1022 [U(1,3A)D]+			425.0852 [U(1,3A)D]-
						409.0907 [M(1,3A-H2O)D]+			407.0757 [M(1,3A-H2O)D]-
						291.0872 [D]+			289.0707 [U]-
						289.0711 [U]+			287.0555 [D]-
						247.0605 [U-C2H2O]+			125.0229 [D(1,4A+2H)]-
						139.0392 [D(1,3A)]+			
						127.0394 [D(1,4A+2H)]+			
						123.0438 [D(1,2B)]+			
**10**	PB IV	12.19	280	579.1500	C30H27O12	427.1014 [U(1,3A)D]+	577.1349	C30H25O12	425.0867 [U(1,3A)D]-
				-0.3		409.0914 [U(1,3-H2O)D]+	0.3		407.0765 [U(1,3A-H2O)D]-
						291.0860 [D]+			289.0711 [D]-
						289.0701 [U]+			245.0811 [U-C2H2O]+
						287.0550 [U(1,3A)D(1,2A-H2O)]+			125.0237 [D(1,4A+2H)]-
						247.0597 [U-C2H2O]+			
						139.0388 [D(1,3A)]+			
						127.0389 [U(1,4A+2H)D]+			
						123.0441 [D(1,2B)]+			
**11**	((Epi)cat)3 (6)	13.20	283	867.2144	C45H39O18	715.1669[U(1,3A)MD]+	865.1988	C45H37O18	713.1475 [U(1,3A)MD]-

				0.8		579.1511 [MD]+<br>409.0907 [M(1,3A-H2O)D]+<br>427.1017 [U(1,3A)D]+<br>409.0919 [M(1,3A-H2O)D]+<br>291.0868 [D]+<br>289.0701 [U]+<br>247.017 [U-C2H2O]+<br>139.0388 [D(1,3A)]+<br>127.0393 [D(1,4A+2H)]+<br>123.0442 [D(1,2B)]+	0.8		577.1346 [MD]-<br>425.0858 [U(1,3A)D]-<br>407.0772 [M(1,3A-H2O)D]-<br>289.0705 [U]-<br>287.0553 [D]-<br>125.0236 [D(1,4A+2H)]-
**12**	PB-gallate-1	14.05	280	731.1599<br>-1.3	C37H31O16	579.1152 [PB+H-Gallic acid]+<br>409.0924 [U(1,3-H2O)D]+<br>291.0868 [D]+<br>287.0546 [U(1,3A)D(1,2A-H2O)]+<br>247.0604 [U-C2H2O]+<br>139.0393 [D(1,3A)]+<br>123.0446 [D(1,2B)]+	729.1398<br>-5.8	C37H29O16	577.1331 [PB-H-Gallic acid]+<br>425.0861 [U(1,3A)D]-<br>407.0756 [U(1,3A-H2O)D]-<br>289.0707 [D]-<br>245.0457 [U-C2H2O]+<br>125.0235 [D(1,4A+2H)]-
**13**	PB-gallate-2	15.14	280	731.1599<br>-1.3	C37H31O16	579.1125[PB+H-Gallic acid]+<br>427.1024 [U(1,3A)D]+<br>409.0923 [U(1,3-H2O)D]+<br>289.0714 [U]+<br>287.0555 [U(1,3A)D(1,2A-H2O)]+<br>247.0611 [U-C2H2O]+<br>139.0393 [D(1,3A)]+	729.1398<br>-5.8	C37H29O16	577.1013 [PB-H-Gallic acid]+<br>425.0869 [U(1,3A)D]-<br>407.0763 [U(1,3A-H2O)D]-<br>289.0705 [D]-<br>245.0455 [U-C2H2O]+<br>125.0234 [D(1,4A+2H)]-

						127.0389 [U(1,4A+2H)D]+<br>123.0446 [D(1,2B)]+			
**14**	Epi	16.31	278	291.0869<br>0.0	C15H15O6	207.0655 [Rup(A)]+<br>147.0443 [0,4B-2H2O]+<br>139.0391 [1,3A]+<br>123.0444 [1,2B]+<br>119.0494 [0,4B-2H2O-CO]+	289.0719<br>0.7	C15H13O6	205.0499 [Rup(A)]-<br>137.0237 [1,3A]-<br>123.0443 [1,3B-CO]-<br>109.0286 [1,3A-CO]-
**15**	((Epi)cat)3 (7)	17.25	283	867.2144<br>0.8	C45H39O18	715.1636 [U(1,3A)MD]+<br>579.1479 [MD]+<br>409.0903[M(1,3A-H2O)D]+<br>427.1001 [U(1,3A)D]+<br>409.0903 [M(1,3A-H2O)D]+<br>291.0859 [D]+<br>289.0701 [U]+<br>247.0596 [U-C2H2O]+<br>139.0387 [D(1,3A)]+<br>127.0391 [D(1,4A+2H)]+<br>123.0442 [D(1,2B)]+	865.1988<br>0.8	C45H37O18	713.1501 [U(1,3A)MD]-<br>577.1334 [MD]-<br>425.0869 [U(1,3A)D]-<br>407.0769 [M(1,3A-H2O)D]-<br>289.0706 [U]-<br>287.0557 [D]-<br>245.0437 [U-C2H2O]+<br>125.0236 [D(1,4A+2H)]-
**16**	PB-gallate-3	19.33	280	731.1599<br>-1.3	C37H31O16	579.1143 [PB+H-Gallic acid]+<br>427.1016 [U(1,3A)D]+<br>409.0914 [U(1,3-H2O)D]+<br>289.0705 [U]+<br>287.0551 [U(1,3A)D(1,2A-H2O)]+<br>247.0596 [U-C2H2O]+<br>139.0387 [D(1,3A)]+	729.1398<br>-5.8	C37H29O16	577.1335 [PB-H-Gallic acid]+<br>425.0869 [U(1,3A)D]-<br>407.0764 [U(1,3A-H2O)D]-<br>289.0710 [D]-<br>245.0447 [U-C2H2O]+<br>125.0233 [D(1,4A+2H)]-

						127.0389 [U(1,4A+2H)D]+<br>123.0440 [D(1,2B)]+			
**17**	PB V	20.37	280	579.1500<br>-0.3	C30H27O12	427.1017 [U(1,3A)D]+<br>409.0924 [U(1,3-H2O)D]+<br>291.0858 [D]+<br>289.0701 [U]+<br>287.0548 [U(1,3A)D(1,2A-H2O)]+<br>247.0597 [U-C2H2O]+<br>139.0390 [D(1,3A)]+<br>127.0388 [U(1,4A+2H)D]+<br>123.0443 [D(1,2B)]+	577.1349<br>0.3	C30H25O12	425.0868 [U(1,3A)D]-<br>407.0765 [U(1,3A-H2O)D]-<br>289.0706 [D]-<br>245.0808 [U-C2H2O]+<br>125.0234 [D(1,4A+2H)]-
**18**	((Epi)cat)3 (8)	20.55	283	867.2144<br>0.8	C45H39O18	715.1688 [U(1,3A)MD]+<br>579.1496 [MD]+<br>409.0920 [M(1,3A-H2O)D]+<br>427.1017 [U(1,3A)D]+<br>409.0920 [M(1,3A-H2O)D]+<br>291.0869 [D]+<br>289.0712 [U]+<br>247.0603 [U-C2H2O]+<br>139.0394 [D(1,3A)]+<br>127.0393 [D(1,4A+2H)]+<br>123.0441 [D(1,2B)]+	865.1988<br>0.8	C45H37O18	713.1500 [U(1,3A)MD]-<br>577.1340 [MD]-<br>425.0862 [U(1,3A)D]-<br>407.0761 [M(1,3A-H2O)D]-<br>289.0705 [U]-<br>287.0551 [D]-<br>245.0436 [U-C2H2O]+<br>125.0231 [D(1,4A+2H)]-
**19**	PB VI	26.17	280	579.1509<br>0.6	C30H27O12	427.1044 [U(1,3A)D]+<br>409.0916 [U(1,3-H2O)D]+<br>291.0870 [D]+	577.1349<br>0.3	C30H25O12	425.0864 [U(1,3A)D]-<br>407.0749 [U(1,3A-H2O)D]-<br>289.0701 [D]-

						289.0712 [U]+<br>287.0560 [U(1,3A)D(1,2A-H2O)]+<br>247.0612 [U-C2H2O]+<br>139.0392 [D(1,3A)]+<br>127.0397 [U(1,4A+2H)D]+<br>123.0443 [D(1,2B)]+			245.0805 [U-C2H2O]+<br>125.0235 [D(1,4A+2H)]-
**20**	PB-gallate-4	29.38	280	731.1599<br>-1.3	C37H31O16	579.1165 [PB+H-Gallic acid]+<br>427.1029 [U(1,3A)D]+<br>409.0916 [U(1,3-H2O)D]+<br>289.0713 [U]+<br>287.0558 [U(1,3A)D(1,2A-H2O)]+<br>247.0597 [U-C2H2O]+<br>139.0387 [D(1,3A)]+<br>127.0389 [U(1,4A+2H)D]+<br>123.0446 [D(1,2B)]+	729.1398<br>-5.8	C37H29O16	577.1031 [PB-H-Gallic acid]+<br>425.0853 [U(1,3A)D]-<br>407.0756[U(1,3A-H2O)D]-<br>289.0704 [D]-<br>245.0444 [U-C2H2O]+<br>125.0234 [D(1,4A+2H)]-
Flavonols									
**21**	Que-hex-hex-1	20.28	264, 344	627.1572<br>1.1	C27H31O17	465.1021 [Y1]+<br>303.0500 [Y0]+	625.137<br>-3.5	C27H29O17	463.0862 [Y1]-<br>301.0342 [Y0]-
**22**	Que-hex-hex-2	24.47	264, 344	627.1562<br>0.1	C27H31O17	465.1035 [Y1]+<br>303.0496 [Y0]+	625.1401<br>-0.4	C27H29O17	463.0878 [Y1]-<br>301.0352 [Y0]-
**23**	Que-hex-hex-3	25.18	264, 344	627.1562<br>0.1	C27H31O17	465.1048 [Y1]+<br>303.0511 [Y0]+	625.1401<br>-0.4	C27H29O17	463.0873 [Y1]-<br>301.0343 [Y0]-
**24**	Que-3-O-gal	27.57	255, 353	n.d.	C21H21O12	487.0854 [M+Na]+<br>303.0511 [Y0]+	463.0824<br>-5.3	C21H19O12	
**25**	Que-3-O-glucoronide	28.11	255, 352	479.0824	C21H19O13	303.0506 [Y0]+	477.0667	C21H17O13	301.0347 [Y0]-

				-0.2		257.0452 [Y0-CHO-OH]+<br>229.0494 [Y0-CHO-OH-CO]+<br>153.0182 [1,3A]+	-0.2		151.0029 [1,3A]-
**26**	Que-3-O-glc	28.33	255, 352	n.d.	C21H21O12	487.0849 [M+Na]+<br>303.0491 [Y0]+	463.0918<br>4.1	C21H19O12	301.0328 [Y0]-<br>271.0233 [Y0-CHO-H]-<br>255.0289 [Y0-CHO-OH]-<br>151.0027 [1,3A]-
**27**	Kaem-3-O-gal	30.15	265, 345	449.1081<br>-0.3	C21H21O11	n.d. [M+Na]+<br>287.0553 [Y0]+	447.0929<br>0.2	C21H19O11	285.0378 [Y0]-<br>151.0025 [1,3A]-
**28**	Kaem-3-O-Glucoronide	31.25	265, 345	463.0878<br>0.1	C21H19O12	n.d. [M+Na]+<br>287.0557 [Y0]+<br>153.0183 [1,3A]+	461.0701<br>-1.9	C21H17O12	285.0390 [Y0]-<br>151.0027 [1,3A]-
**29**	Kaem-3-O-glc	31.43	265, 348	449.1080<br>-0.4	C21H21O11	n.d. [M+Na]+<br>287.0558[Y0]+	447.0934<br>0.7	C21H19O11	285.0377 [Y0]-<br>151.0027 [1,3A]-
**30**	Iso-3-O-gal	31.77	254, 352	479.1192<br>0.2	C22H23O12	317.0663 [Y0]+	477.1033<br>0.0	C22H21O12	477.1033 [M-H]-<br>315.0447 [Y0]-
**31**	Iso-3-O-glc	32.32	254, 352	479.1188<br>-0.2	C22H23O12	317.0663 [Y0]+	477.1042<br>0.9	C22H21O12	477.1033 [M-H]-<br>315.0447 [Y0]-
Hydroxycinnamic acids									
**32**	pCumhex	10.58	313	n.d.			325.0925<br>0.2	C15H17O8	163.0392 [Y0]-<br>119.0491 [Y0-CO2]-
Hydroxybenzoic acid									
**33**	Gal acid	1.92	271	171.0301			169.0107<br>-3.0	C7H5O5	125.0232 [M-H-CO2]-<br>107.0132 [M-H-H2O-CO2]-<br>79.0178 [M+H-CO2-H2O-CO]-

**34**	Galloyl Derivative	3.60	279	Unknown	153.0199 [Y0-H2O]+<br>125.0236 [Y0-H2O-CO]+<br>109.0290 [Y0-H2O-CO2]+<br>107.0136 [Y0-2H2O-CO]+<br>81.0345 [Y0-CO2-H2O-CO]+	Unknown	169.0154 [Y0]-<br>79.0181 [Y0-CO2-H2O-CO]-
